# Baseline and 24-Hour Changes in the Frontal QRS-T Angle During Non-Invasive Ventilation and Their Association with Clinical Outcomes in Acute Hypercapnic Respiratory Failure

**DOI:** 10.3390/jcm15187066

**Published:** 2026-09-11

**Authors:** Murat Karamanlıoğlu, Murat Yıldız, Oral Menteş, Suzan Şahan, Maşide Arı, Suna Kavurgacı, Deniz Çelik, Ezgi Gürel Akan

**Affiliations:** 1 Department of Cardiology, Ankara Atatürk Sanatorium Training and Research Hospital, University of Health Sciences, Ankara 06280, Türkiye; 2 Department of Chest Diseases, Ankara Atatürk Sanatorium Training and Research Hospital, University of Health Sciences, Ankara 06280, Türkiye; drmuratyildiz85@gmail.com (M.Y.); suna.dr01@gmail.com (S.K.); gurelezgi@gmail.com (E.G.A.); 3 Department of Intensive Care, Ankara Atatürk Sanatorium Training and Research Hospital, University of Health Sciences, Ankara 06280, Türkiye; omentes@live.com (O.M.); masidetuten@icloud.com (M.A.); 4 Department of Cardiology, Ankara Çubuk State Hospital, Ankara 06760, Türkiye; suzan_sahan@hotmail.com; 5 Department of Chest Diseases, Faculty of Medicine, Alanya Alaaddin Keykubat University, Alanya 07425, Türkiye; deniz.celik@alanya.edu.tr

**Keywords:** acute hypercapnic respiratory failure, frontal QRS-T angle, non-invasive mechanical ventilation, BPAP, electrocardiography, mortality

## Abstract

**Background/Objectives**: Acute hypercapnic respiratory failure is associated with significant morbidity and mortality despite advances in non-invasive mechanical ventilation (NIMV) therapy. The frontal QRS-T angle is a simple electrocardiographic marker reflecting ventricular depolarization–repolarization heterogeneity and has been associated with adverse cardiovascular outcomes. However, the relationship between dynamic changes in frontal QRS-T angle and NIMV treatment response in acute hypercapnic respiratory failure remains unclear. This study aimed to evaluate the association between frontal QRS-T angle changes after NIMV treatment and clinical outcomes in patients with acute hypercapnic respiratory failure. **Methods**: This retrospective observational cohort study included 186 adult patients hospitalized with acute hypercapnic respiratory failure and treated with bilevel positive airway pressure between January 2024 and December 2025. Frontal QRS-T angle values were obtained from standard 12-lead electrocardiograms recorded before NIMV initiation (T0) and at the 24th hour of treatment (T24). Clinical, laboratory, arterial blood gas, and electrocardiographic parameters were analyzed. Receiver operating characteristic (ROC) analysis, Kaplan–Meier survival analysis, correlation analysis, and multivariable logistic regression analysis were performed to evaluate the prognostic significance of frontal QRS-T angle. **Results**: Successful NIMV treatment was achieved in 143 patients (76.9%), whereas 43 patients (23.1%) experienced NIMV failure. Patients with failed NIMV treatment had significantly higher baseline frontal QRS-T angle values compared with the successful group [128° (94–156) vs. 71° (48–101), *p* < 0.001]. Frontal QRS-T angle significantly decreased after treatment in the successful NIMV group but not in the failed group. A significant positive correlation was observed between ΔPaCO_2_ and Δfrontal QRS-T angle (r = 0.624, *p* < 0.001). ROC analysis demonstrated that baseline frontal QRS-T angle predicted NIMV failure with an AUC of 0.842 (95% CI: 0.773–0.898) and in-hospital mortality with an AUC of 0.811 (95% CI: 0.735–0.873). Kaplan–Meier analysis showed significantly lower survival rates in patients with frontal QRS-T angle >90° (log-rank *p* < 0.001). Multivariable logistic regression analysis identified frontal QRS-T angle >90° as an independent predictor of in-hospital mortality (OR: 3.617, 95% CI: 1.484–8.813, *p* = 0.005). **Conclusions**: Baseline frontal QRS-T angle was associated with NIMV failure and in-hospital mortality, while its 24-h change was associated with physiological response among patients with paired T0–T24 measurements. These findings are hypothesis-generating and do not establish the utility of frontal QRS-T angle monitoring for immediate escalation or intubation decisions.

## 1. Introduction

Acute hypercapnic respiratory failure is a clinically important form of respiratory failure characterized by alveolar hypoventilation, elevated arterial carbon dioxide tension, and, frequently, respiratory acidosis [1]. It is most commonly encountered in patients with acute exacerbation of chronic obstructive pulmonary disease, but it may also occur in obesity hypoventilation syndrome, bronchiectasis, pneumonia, neuromuscular disorders, and advanced chronic respiratory diseases [2,3]. The accumulation of carbon dioxide and the accompanying reduction in pH may rapidly impair respiratory muscle performance, alter pulmonary vascular tone, and contribute to systemic hemodynamic instability. Therefore, early recognition of treatment response is essential in order to prevent clinical deterioration, endotracheal intubation, and increased mortality [4,5].

Non-invasive mechanical ventilation (NIMV), particularly bilevel positive airway pressure, is a cornerstone treatment for acute hypercapnic respiratory failure [6,7]. By improving alveolar ventilation, reducing the work of breathing, increasing pH, and decreasing PaCO_2_, non-invasive ventilation can reverse respiratory acidosis and reduce the need for invasive mechanical ventilation [8,9]. International recommendations support the use of bilevel non-invasive ventilation in patients with acute hypercapnic respiratory failure, especially in COPD exacerbations with respiratory acidosis, because it decreases treatment failure, intubation risk, and mortality when applied appropriately [10,11]. Nevertheless, NIMV failure remains a clinically relevant problem. Delayed recognition of NIMV failure may expose patients to prolonged respiratory distress and delayed intubation, both of which are associated with worse outcomes [10,12]. For this reason, easily obtainable parameters that reflect early physiological improvement during NIMV may provide additional value in bedside risk stratification.

The cardiovascular consequences of acute hypercapnic respiratory failure are increasingly recognized. Hypoxemia and hypercapnia can induce pulmonary vasoconstriction, increase pulmonary arterial pressure, and augment right ventricular afterload [13,14,15]. In patients with chronic lung disease, these acute changes may occur on the background of pre-existing pulmonary hypertension, right ventricular strain, or subclinical myocardial dysfunction [16,17]. As a result, respiratory failure may not only disturb gas exchange but may also affect cardiac electrophysiology through changes in ventricular loading conditions, autonomic tone, acid–base balance, and myocardial repolarization [18,19]. These pathophysiological mechanisms suggest that electrocardiographic markers may reflect systemic and cardiopulmonary stress during acute hypercapnic episodes.

The frontal QRS-T angle is a simple electrocardiographic parameter that represents the spatial difference between ventricular depolarization and repolarization vectors in the frontal plane [20,21,22,23]. On a standard 12-lead ECG, the frontal QRS-T angle can be obtained from the automatically reported frontal QRS axis and T-wave axis. It is calculated as the absolute difference between these two axes; when this difference exceeds 180°, the complementary angle is used by subtracting the absolute difference from 360°, thereby yielding a value between 0° and 180°. Serial measurements also permit calculation of the change in frontal QRS-T angle, with negative values indicating narrowing and positive values indicating widening of the angle [24,25,26]. A widened frontal QRS-T angle indicates increased heterogeneity between depolarization and repolarization, which may reflect electrical remodeling, myocardial strain, ischemia, ventricular dysfunction, or altered autonomic regulation [21,27,28]. Previous studies have shown that an abnormal frontal QRS-T angle is associated with adverse cardiovascular events and all-cause mortality in different clinical populations [29,30,31]. Because the frontal QRS-T angle can be calculated from standard 12-lead ECG recordings, it has practical advantages as a non-invasive, inexpensive, and widely available marker.

Despite the growing evidence supporting the prognostic value of frontal QRS-T angle in cardiovascular diseases, its role in acute respiratory failure remains insufficiently investigated. In acute hypercapnic respiratory failure, improvement after NIMV is traditionally assessed using clinical parameters such as respiratory rate, mental status, accessory muscle use, oxygenation, pH, and PaCO_2_ [24,25,26]. However, these parameters may not fully capture the cardiopulmonary interaction occurring during treatment. If NIMV successfully reduces hypercapnia and respiratory acidosis, it may also decrease pulmonary vasoconstriction, right ventricular strain, and repolarization abnormalities. Therefore, dynamic changes in the frontal QRS-T angle before and after NIMV may provide an additional electrophysiological marker of treatment response.

In this context, evaluating the change in frontal QRS-T angle between baseline and the 24th hour of NIMV treatment may help identify patients who respond favorably to non-invasive ventilation. A decrease in frontal QRS-T angle after NIMV may reflect improvement in ventricular electrical heterogeneity secondary to better gas exchange, reduced PaCO_2_, correction of acidosis, and decreased cardiopulmonary stress. Conversely, persistence or widening of the frontal QRS-T angle may indicate ongoing physiological stress and may be associated with NIMV failure, need for invasive mechanical ventilation, prolonged intensive care stay, or mortality.

Therefore, this study aimed to evaluate the change in frontal QRS-T angle measured from 12-lead ECG recordings before NIMV and at the 24th hour of NIMV treatment in adult patients with acute hypercapnic respiratory failure. In addition, the study seeks to investigate whether this change is associated with clinical and biochemical response to NIMV, including improvement in pH, reduction in PaCO_2_, absence of intubation requirement, and treatment success. By focusing on baseline and 24-h measurements of a readily available ECG-derived parameter, this study aimed to evaluate whether the frontal QRS-T angle and its change over the first 24 h are associated with physiological response and clinical outcomes during NIMV. The study was not designed to assess immediate ECG changes during the first hours of treatment or to guide early intubation decisions.

## 2. Materials and Methods

### 2.1. Study Design

This retrospective observational cohort study was conducted in the Level II Chest Diseases Intensive Care Unit of Ankara Atatürk Sanatorium Training and Research Hospital, Health Sciences University, Ankara, Türkiye. Adult patients hospitalized with acute hypercapnic respiratory failure between 1 January 2024, and 31 December 2025, were retrospectively evaluated through the hospital electronic medical record system. The study protocol was reviewed and approved by the Scientific Research Ethics Committee of Ankara Atatürk Sanatorium Training and Research Hospital (Decision No: 2024-BÇEK/518; Date: 8 April 2026).

The study was conducted in accordance with the ethical principles of the Declaration of Helsinki. Due to the retrospective nature of the study, no additional intervention, laboratory testing, or electrocardiographic examination was performed for research purposes. All data were obtained from routinely recorded clinical follow-up files and anonymized before statistical analysis.

### 2.2. Study Population, Inclusion Criteria, and Exclusion Criteria

Patients diagnosed with acute hypercapnic respiratory failure and treated with non-invasive mechanical ventilation (NIMV), specifically bilevel positive airway pressure (BPAP), were screened for eligibility. Acute hypercapnic respiratory failure was defined as the presence of arterial partial carbon dioxide pressure (PaCO_2_) ≥45 mmHg together with clinical findings consistent with hypercapnic respiratory failure, including dyspnea, respiratory distress, altered mental status, tachypnea, or use of accessory respiratory muscles [4,10].

Adult patients aged ≥ 18 years who were hospitalized with a diagnosis of acute hypercapnic respiratory failure and received BPAP treatment during hospitalization were included in the study. Acute hypercapnic respiratory failure was defined as the presence of arterial PaCO_2_ elevation accompanied by clinical findings consistent with respiratory failure. Eligible patients were required to have two standard 12-lead electrocardiographic recordings available, including one obtained immediately before BPAP initiation (T0) and another recorded at the 24th ± 2 h of treatment (T24). In addition, arterial blood gas analyses corresponding to both time points had to be available. Only patients who were followed in the intensive care unit with routine daily ECG monitoring as part of standard clinical practice and who had complete clinical outcome data, including need for endotracheal intubation, intensive care unit length of stay, and in-hospital mortality, were included in the final analysis. For the dynamic analysis, both the T24 ECG and the corresponding arterial blood gas assessment were required to have been obtained before endotracheal intubation. Patients intubated before completion of the scheduled T24 assessment were not eligible because a valid pre-intubation T24 ECG could not be obtained.

Patients were excluded if they had permanent pacemaker implantation or cardiac resynchronization therapy, left bundle branch block, or QRS duration ≥ 120 ms on baseline or follow-up ECG. Patients diagnosed with acute ST-segment elevation myocardial infarction at admission or within the first 24 h were also excluded. Additional exclusion criteria included severe electrolyte abnormalities, such as serum potassium <3.0 or >5.5 mmol/L, clinically significant hypomagnesemia or hypocalcemia, development of rhythm change between T0 and T24 ECG recordings (e.g., conversion from sinus rhythm to atrial fibrillation), the requirement for immediate endotracheal intubation at admission or intubation before acquisition of the scheduled pre-intubation T24 ECG and arterial blood gas assessment, death within the first 24 h before acquisition of the T24 ECG, and missing clinical, laboratory, or electrocardiographic data required for statistical analysis.

### 2.3. Definition of NIMV Success

NIMV success was defined according to the early clinical and biochemical response observed within the first 24 h of treatment. Patients were classified as having successful NIMV treatment when at least two of the following criteria were achieved at the 24th hour of therapy: an increase in arterial pH of at least 0.03, a decrease in PaCO_2_ of at least 5 mmHg, and absence of the need for invasive mechanical ventilation. Patients who failed to meet these criteria or required endotracheal intubation during follow-up were classified as having NIMV failure.

### 2.4. Electrocardiographic Evaluation

Standard 12-lead ECG recordings were obtained using the same hospital ECG system at a paper speed of 25 mm/s and a calibration of 10 mm/mV. The frontal-plane QRS axis and T-wave axis were automatically generated by the ECG device and recorded in degrees. For each ECG, the raw angular difference (d) was calculated as the absolute difference between the QRS axis and T-wave axis: d = |QRS axis − T-wave axis|. When d was ≤180°, the frontal QRS-T angle was equal to d; when d exceeded 180°, the frontal QRS-T angle was calculated as 360° − d. Accordingly, all frontal QRS-T angle values ranged from 0° to 180° [32,33].

**Figure 1:**
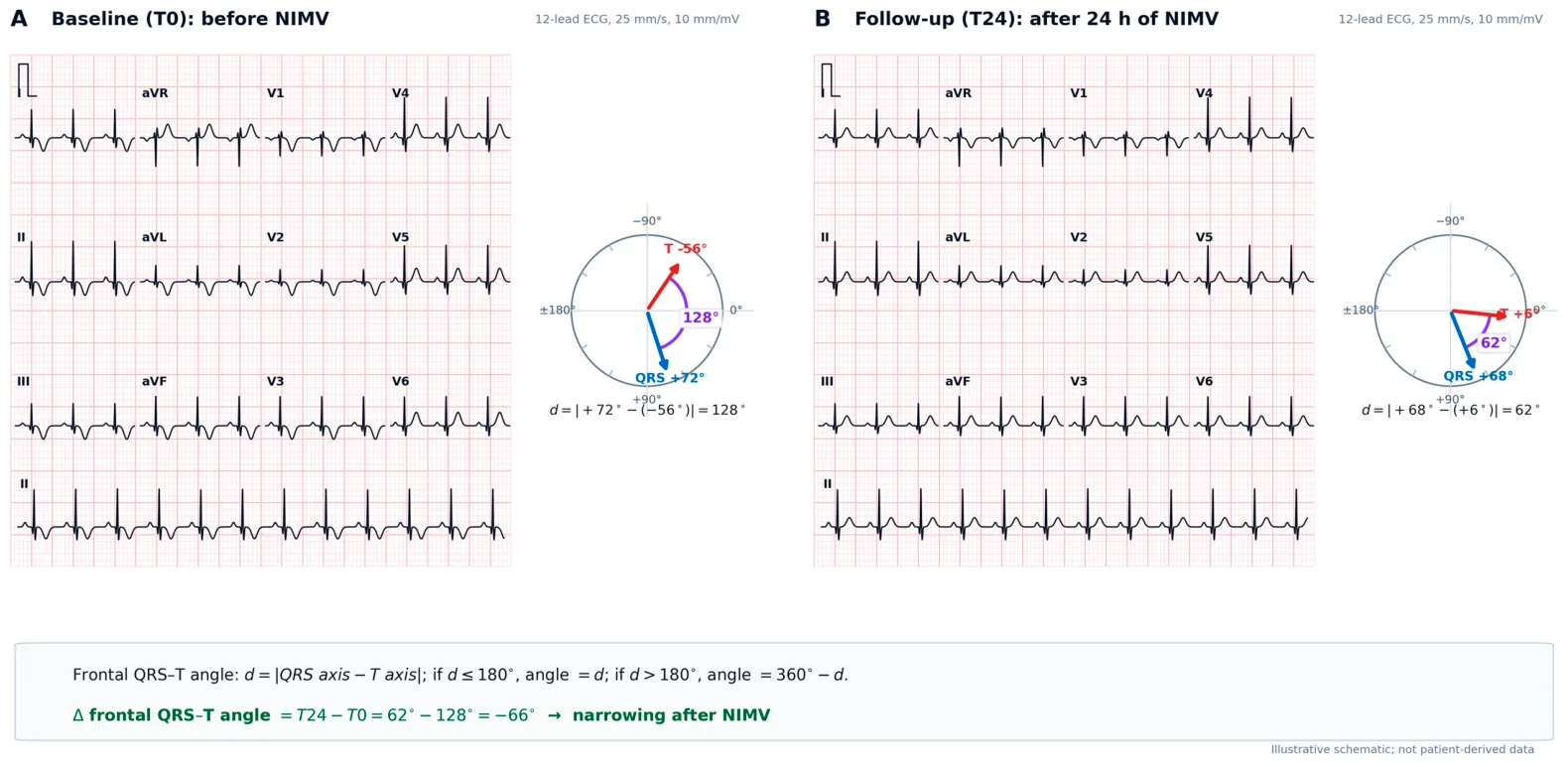
Calculation of the frontal QRS-T angle and its change during NIMV treatment. The frontal QRS-T angle was calculated from the automatically reported frontal QRS axis and T-wave axis on standard 12-lead ECG recordings. The absolute difference between the two axes was used when it was ≤180°; when the difference exceeded 180°, the angle was calculated as 360° minus the absolute difference. Δ frontal QRS-T angle was calculated as the T24 value minus the T0 value. Negative values indicate narrowing, whereas positive values indicate widening of the angle.

Frontal QRS-T angles were calculated separately from the ECGs obtained immediately before BPAP initiation (T0) and at the 24th ± 2 h of treatment (T24). The change in frontal QRS-T angle was defined as Δ frontal QRS-T angle = T24 frontal QRS-T angle − T0 frontal QRS-T angle. Thus, a negative Δ value indicated narrowing of the angle after NIMV, whereas a positive value indicated widening. Representative de-identified ECG recordings and the corresponding calculation procedure are presented in Figure 1.

Patients were additionally categorized into low and high frontal QRS-T angle groups according to previously reported prognostic threshold values in the literature [34].

### 2.5. Data Collection and Clinical Outcomes

The following demographic, clinical, laboratory, electrocardiographic, and outcome variables were retrospectively collected from electronic hospital records and patient follow-up files. Demographic variables included age, sex, hospital admission date, and protocol number. Clinical data consisted of underlying respiratory diseases, including chronic obstructive pulmonary disease, pneumonia, bronchiectasis, interstitial lung disease, lung malignancy, chronic respiratory failure, and other pulmonary disorders. In addition, accompanying comorbid conditions such as diabetes mellitus, hypertension, chronic kidney disease, heart failure, coronary artery disease, and other systemic diseases were recorded.

Laboratory parameters obtained at hospital admission or within the first 24 h of hospitalization included arterial blood gas measurements and routinely evaluated biochemical markers. These parameters comprised arterial pH, PaCO_2_, PaO_2_, bicarbonate levels, C-reactive protein, serum albumin, glucose, complete blood count parameters, lymphocyte percentage, and other routinely measured laboratory variables. Electrocardiographic data, including frontal QRS-T angle measurements obtained from standard 12-lead ECG recordings before and after NIMV treatment, were also analyzed.

Clinical outcomes evaluated in the study included the need for invasive mechanical ventilation, duration of intensive care unit stay, total length of hospitalization, in-hospital mortality, and time to mortality during hospitalization. All collected data were anonymized before statistical analysis, and no additional intervention or laboratory testing was performed for study purposes because of the retrospective design of the study. Time to endotracheal intubation was calculated from the initiation of NIMV to endotracheal tube placement. For descriptive analysis, intubation timing was categorized as ≤24 h, >24–48 h, >48–72 h, and >72 h. The scheduled T24 ECG and arterial blood gas assessments were required to have been completed before intubation for inclusion in the paired T0–T24 analysis.

### 2.6. Statistical Analysis

All statistical analyses were performed using IBM SPSS Statistics for Windows version 27.0 (IBM Corp., Armonk, NY, USA) and MedCalc Statistical Software version 20.104 (MedCalc Software Ltd., Ostend, Belgium). The distribution characteristics of continuous variables were assessed using the Kolmogorov–Smirnov and Shapiro–Wilk normality tests. In addition to formal normality testing, histogram distributions, skewness-kurtosis values, and outlier analyses were evaluated to determine data distribution patterns. Categorical variables were expressed as numbers and percentages [*n* (%)], whereas continuous variables with normal distribution were presented as mean ± standard deviation. Non-normally distributed variables were expressed as median and interquartile range (IQR).

Comparisons between two independent groups were performed using Student’s *t*-test for normally distributed continuous variables and Mann–Whitney U test for non-normally distributed variables. Categorical variables were compared using the Chi-square test or Fisher’s exact test where appropriate. Paired comparisons between pre-treatment and post-treatment frontal QRS-T angle measurements were analyzed using paired-samples *t*-test for normally distributed data and Wilcoxon signed-rank test for non-parametric data.

Correlation analyses between changes in PaCO_2_ levels and frontal QRS-T angle values were evaluated using Pearson correlation analysis when both variables demonstrated normal distribution and Spearman rank correlation analysis when at least one variable was non-normally distributed. Receiver operating characteristic (ROC) curve analysis was performed to evaluate the discriminatory performance of baseline frontal QRS-T angle, Δ frontal QRS-T angle, baseline pH, PaCO_2_, and lactate for NIMV failure and in-hospital mortality. AUCs with 95% confidence intervals were calculated. Optimal cut-off values were determined using the Youden index, and the corresponding sensitivity, specificity, positive predictive value, and negative predictive value were reported. Because lower pH values indicated greater risk, the test direction for pH was reversed when constructing the ROC curves. Pairwise comparisons of correlated AUCs were performed using DeLong’s test, with the baseline frontal QRS-T angle used as the reference marker.

Kaplan–Meier survival analysis and log-rank testing were used to compare survival distributions between patients with low and high frontal QRS-T angle values. To identify independent predictors of mortality, multivariable logistic regression analysis was performed including clinically relevant variables potentially associated with mortality in acute hypercapnic respiratory failure. A backward stepwise elimination method was applied to establish the final regression model with the highest explanatory performance. Adjusted odds ratios (ORs) with 95% confidence intervals (CIs) were reported, and model performance was evaluated using Nagelkerke R^2^ values. A two-tailed *p*-value <0.05 was considered statistically significant for all analyses.

## 3. Results

**Figure 2:**
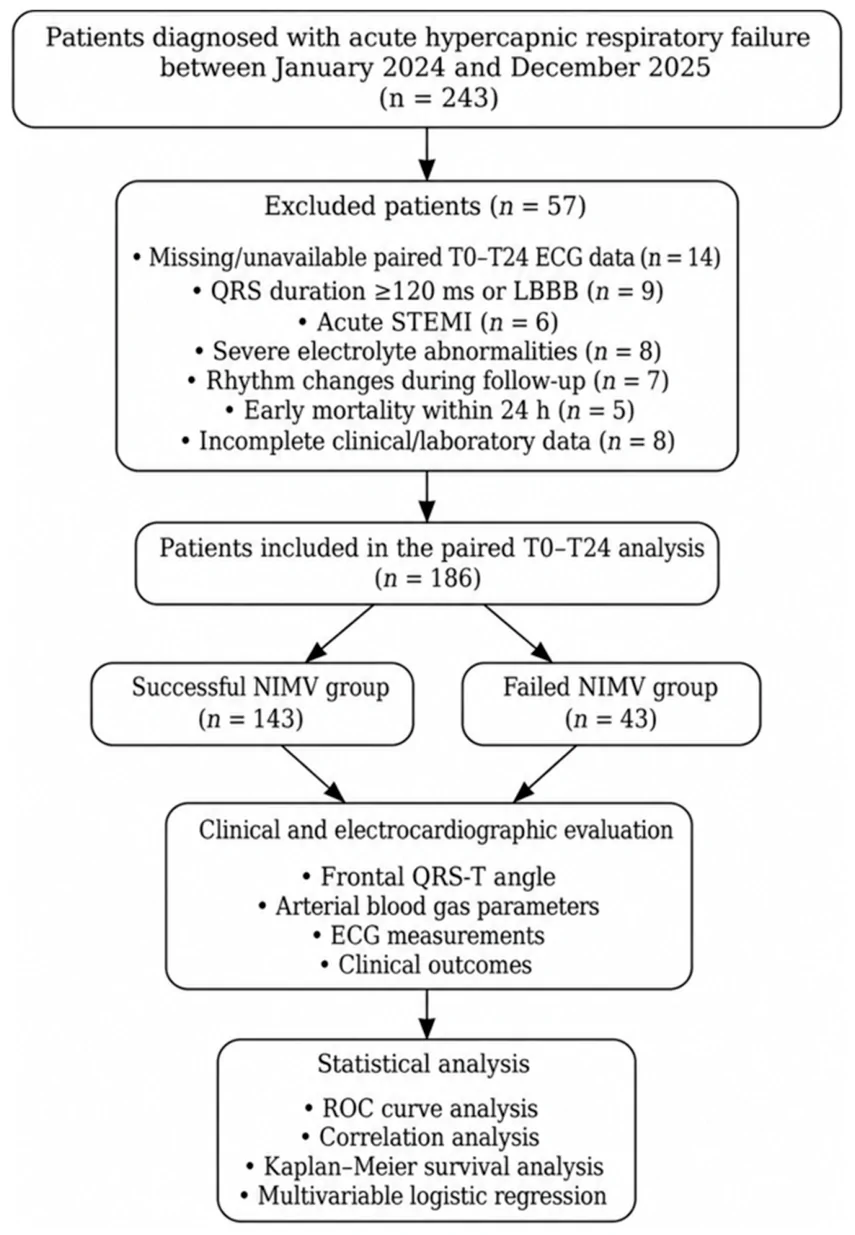
Flowchart of the study.

A total of 243 patients diagnosed with acute hypercapnic respiratory failure and treated with BPAP/NIMV between January 2024 and December 2025 were screened for eligibility. After excluding patients with missing electrocardiographic data, rhythm abnormalities, severe electrolyte disturbances, acute myocardial infarction, early mortality, and other predefined exclusion criteria, 186 patients were included in the final analysis. Among the included patients, 143 (76.9%) were classified as successful NIMV responders, whereas 43 (23.1%) were classified as NIMV failures according to predefined clinical and biochemical response criteria. Among the 37 patients who required invasive mechanical ventilation, the median interval from NIMV initiation to endotracheal intubation was 32 h (IQR, 28–46 h). All scheduled T24 ECG and arterial blood gas assessments were completed before endotracheal intubation; therefore, none of the patients included in the paired T0–T24 analysis underwent intubation before the T24 assessment (Figure 2).

The study population consisted of 186 patients with a mean age of 69.4 ± 11.8 years, of whom 118 (63.4%) were male. Chronic obstructive pulmonary disease was the most common underlying respiratory disease, observed in 121 patients (65.1%), followed by chronic respiratory failure in 47 patients (25.3%) and pneumonia in 38 patients (20.4%). Hypertension and diabetes mellitus were the most frequent comorbid diseases, present in 102 (54.8%) and 74 (39.8%) patients, respectively. At admission, patients demonstrated significant respiratory compromise with a mean respiratory rate of 29 ± 6 breaths/min, oxygen saturation of 82 ± 8%, and heart rate of 104 ± 18 beats/min. The median intensive care unit stay was 6 (4–11) days, while the median total hospitalization duration was 11 (7–17) days. Successful NIMV treatment was achieved in 143 patients (76.9%), whereas 43 patients (23.1%) experienced NIMV failure. Invasive mechanical ventilation was required in 37 patients (19.9%), and in-hospital mortality occurred in 29 patients (15.6%) (Table 1).

Baseline arterial blood gas analysis demonstrated significant hypercapnic respiratory failure with a mean pH of 7.28 ± 0.07, PaCO_2_ of 68.9 ± 14.6 mmHg, and PaO_2_ of 58.4 ± 12.7 mmHg. Mean bicarbonate and lactate levels were 31.6 ± 6.5 mmol/L and 2.3 ± 1.1 mmol/L, respectively. Laboratory evaluation revealed a mean white blood cell count of 11.8 ± 4.7 ×10^3^/µL, hemoglobin level of 13.1 ± 2.0 g/dL, lymphocyte percentage of 15.4 ± 8.2%, platelet count of 256 ± 88 ×10^3^/µL, serum albumin level of 3.4 ± 0.6 g/dL, glucose level of 162 ± 58 mg/dL, and creatinine level of 1.12 ± 0.48 mg/dL. Median C-reactive protein level was 48 (22–96) mg/L. Electrocardiographic evaluation showed sinus rhythm in 169 patients (90.9%), with a mean ECG heart rate of 101 ± 17 beats/min, QRS duration of 96 ± 11 ms, and QTc interval of 438 ± 29 ms. The median baseline frontal QRS-T angle was 82° (54–118), and 79 patients (42.5%) had a frontal QRS-T angle greater than 90° (Table 2).

Patients with failed NIMV treatment were significantly older than those with successful treatment (74.2 ± 12.4 vs. 67.8 ± 10.9 years, *p* = 0.002). Pneumonia was more frequent in the failed NIMV group compared with the successful group [14 (32.6%) vs. 24 (16.8%), *p* = 0.024]. Similarly, coronary artery disease, heart failure, and chronic kidney disease were significantly more common among patients with failed NIMV treatment (*p* = 0.045, *p* = 0.011, and *p* = 0.014, respectively). At admission, patients in the failed NIMV group demonstrated significantly higher heart rate (112 ± 19 vs. 101 ± 17 beats/min, *p* = 0.001) and respiratory rate (32 ± 7 vs. 28 ± 5 breaths/min, *p* < 0.001), whereas oxygen saturation levels were significantly lower compared with the successful NIMV group (76 ± 8% vs. 84 ± 7%, *p* < 0.001). No statistically significant differences were observed between the groups regarding sex distribution, body mass index, smoking status, COPD frequency, bronchiectasis, interstitial lung disease, lung malignancy, obesity hypoventilation syndrome, hypertension, or diabetes mellitus (all *p* > 0.05) (Table 3).

Patients with failed NIMV treatment demonstrated significantly more severe respiratory acidosis and hypercapnia at admission compared with the successful NIMV group. Baseline pH values were significantly lower in the failed NIMV group (7.22 ± 0.08 vs. 7.30 ± 0.06, *p* < 0.001), whereas PaCO_2_ levels were markedly higher (80.1 ± 16.3 vs. 65.4 ± 12.2 mmHg, *p* < 0.001). Patients with NIMV failure also had significantly lower PaO_2_ levels (52.1 ± 13.5 vs. 60.2 ± 11.4 mmHg, *p* < 0.001), higher bicarbonate concentrations (34.1 ± 7.2 vs. 30.8 ± 5.8 mmol/L, *p* = 0.003), and elevated lactate levels (3.1 ± 1.4 vs. 2.0 ± 0.9 mmol/L, *p* < 0.001). Laboratory analyses revealed significantly higher white blood cell count, C-reactive protein, glucose, and creatinine levels in the failed NIMV group, whereas hemoglobin, lymphocyte percentage, and serum albumin levels were significantly lower (all *p* < 0.05). Electrocardiographic evaluation demonstrated significantly higher ECG heart rate, QRS duration, QTc interval, and baseline frontal QRS-T angle values among patients with failed NIMV treatment (all *p* ≤ 0.002). The proportion of patients with frontal QRS-T angle >90° was significantly greater in the failed NIMV group compared with the successful group [33 (76.7%) vs. 46 (32.2%), *p* < 0.001]. Furthermore, the reduction in frontal QRS-T angle after treatment was significantly smaller in patients with NIMV failure [−5 (−12 to 4) vs. −24 (−41 to −10), *p* < 0.001]. Clinical outcomes were markedly worse in the failed NIMV group. Invasive mechanical ventilation was required in 37 patients (86.0%) in the failed NIMV group, whereas none of the successfully treated patients required intubation (*p* < 0.001). The median interval from NIMV initiation to endotracheal intubation among these 37 patients was 32 h (IQR, 28–46 h). None underwent intubation within the first 24 h or before completion of the scheduled T24 ECG and arterial blood gas assessments. Twenty-nine patients (78.4%) were intubated between 24 and 48 h, five (13.5%) between 48 and 72 h, and three (8.1%) after 72 h. Patients with NIMV failure also had significantly longer intensive care unit stay [14 (9–21) vs. 5 (3–8) days, *p* < 0.001], longer total hospitalization duration [19 (12–28) vs. 9 (6–13) days, *p* < 0.001], and higher in-hospital mortality rates [20 (46.5%) vs. 9 (6.3%), *p* < 0.001] (Table 4).

In the overall study population, frontal QRS-T angle values significantly decreased after 24 h of BPAP/NIMV treatment, from 82° (54–118) at baseline to 61° (39–92) after treatment (*p* < 0.001). Similarly, significant reductions were observed in ECG heart rate (101 ± 17 vs. 92 ± 15 beats/min, *p* < 0.001), QRS duration (96 ± 11 vs. 94 ± 10 ms, *p* = 0.041), and QTc interval (438 ± 29 vs. 425 ± 24 ms, *p* < 0.001). In the successful NIMV group, frontal QRS-T angle demonstrated a marked reduction from 71° (48–101) to 44° (28–73) following treatment (*p* < 0.001). Significant decreases were also observed in heart rate on ECG (99 ± 16 vs. 88 ± 13 beats/min, *p* < 0.001), QRS duration (95 ± 10 vs. 93 ± 9 ms, *p* = 0.032), and QTc interval (432 ± 26 vs. 419 ± 21 ms, *p* < 0.001). In contrast, patients with failed NIMV treatment did not demonstrate statistically significant changes in frontal QRS-T angle [128° (94–156) vs. 119° (88–149), *p* = 0.071], ECG heart rate (110 ± 18 vs. 105 ± 17 beats/min, *p* = 0.084), QRS duration (101 ± 13 vs. 100 ± 12 ms, *p* = 0.412), or QTc interval (457 ± 34 vs. 451 ± 31 ms, *p* = 0.118) between baseline and post-treatment measurements (Table 5).

Correlation analysis demonstrated a significant positive association between the reduction in PaCO_2_ levels and the decrease in frontal QRS-T angle values following NIMV treatment (r = 0.624, *p* < 0.001). Significant positive correlations were also observed between ΔPaCO_2_ and ΔQTc interval (r = 0.381, *p* < 0.001), as well as between ΔPaCO_2_ and Δheart rate (r = 0.294, *p* = 0.002). In contrast, ΔpH showed a significant negative correlation with Δfrontal QRS-T angle (r = −0.517, *p* < 0.001). A weaker but statistically significant negative correlation was identified between ΔPaO_2_ and Δfrontal QRS-T angle (r = −0.248, *p* = 0.011). Additionally, Δlactate levels demonstrated a significant positive correlation with Δfrontal QRS-T angle values (r = 0.336, *p* < 0.001) (Table 6).

**Figure 3:**
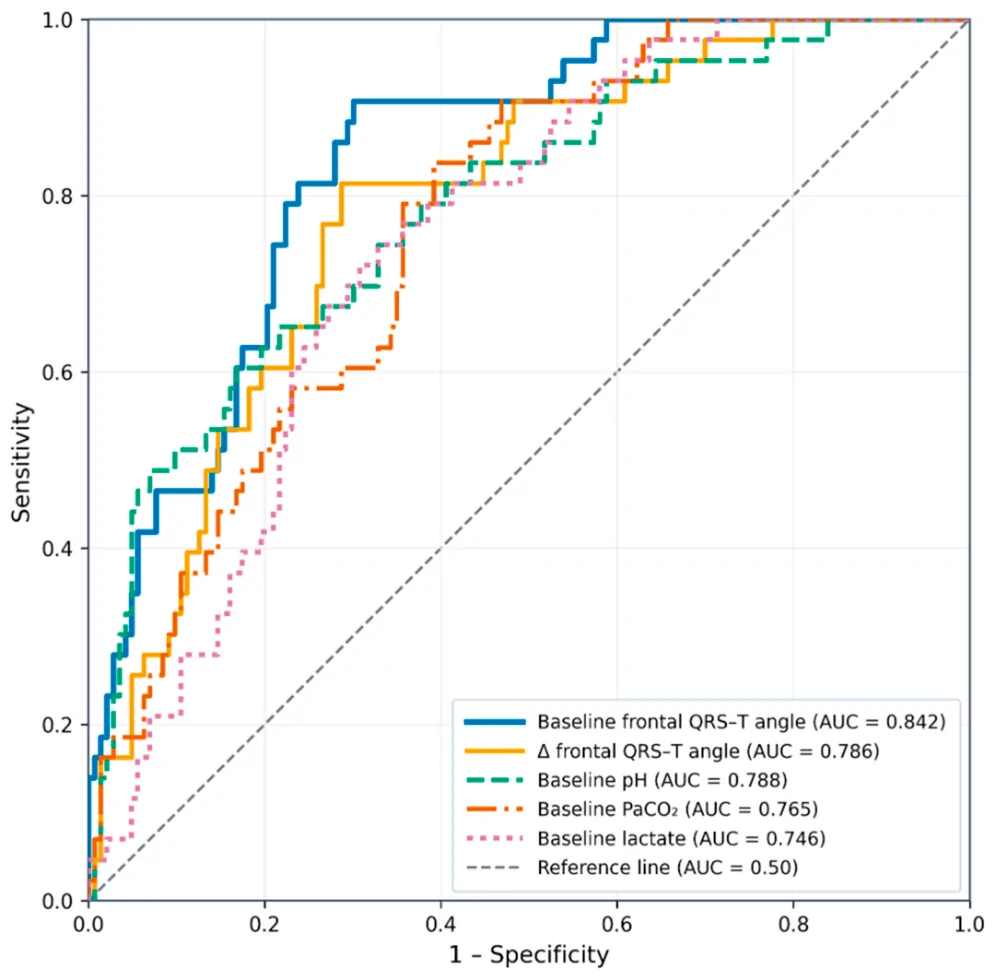
Comparative receiver operating characteristic curves of baseline frontal QRS-T angle, Δ frontal QRS-T angle, baseline pH, PaCO_2_, and lactate for predicting NIMV failure.

**Figure 4:**
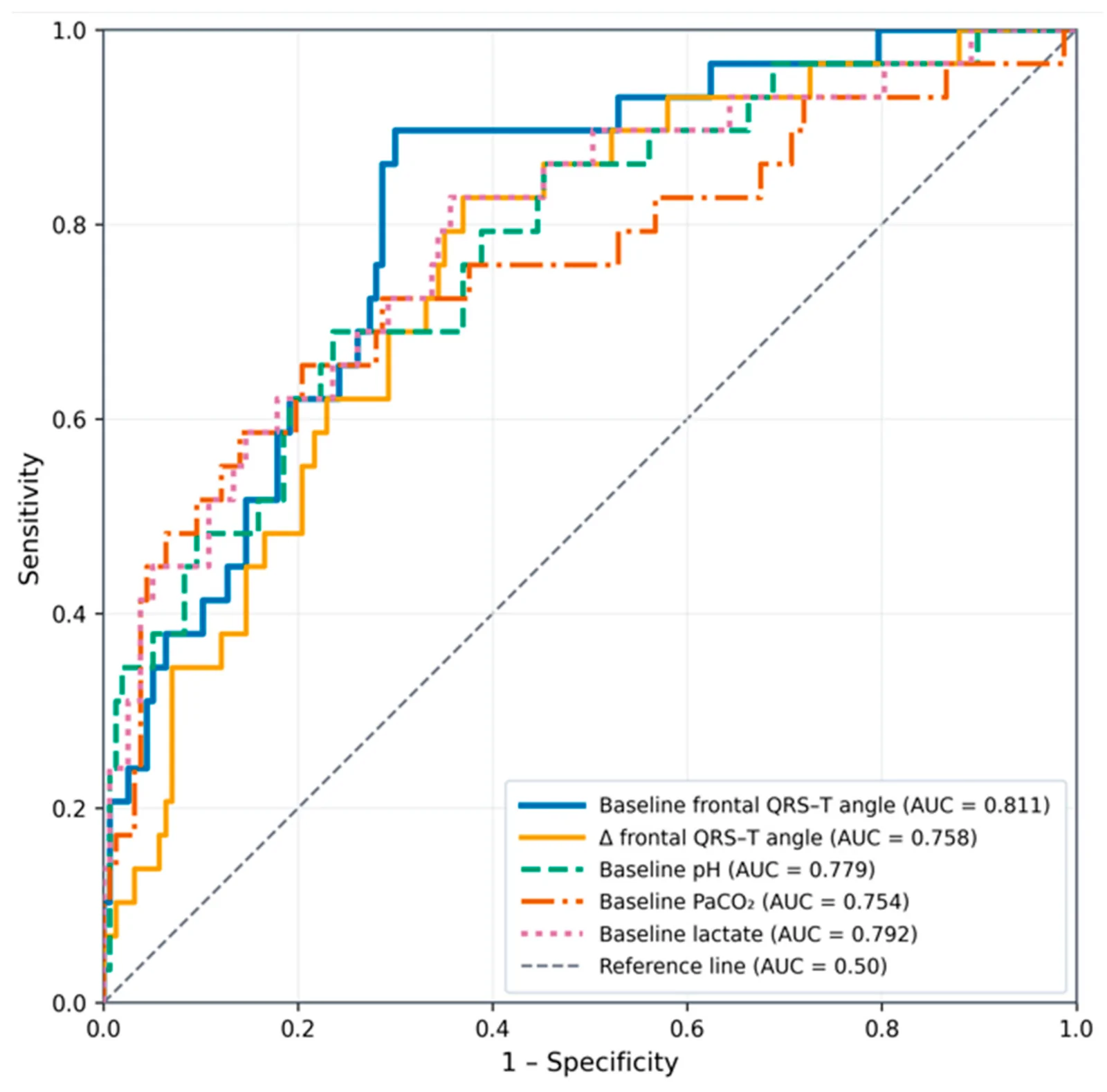
Comparative receiver operating characteristic curves of baseline frontal QRS-T angle, Δ frontal QRS-T angle, baseline pH, PaCO_2_, and lactate for predicting in-hospital mortality.

Comparative ROC analysis showed that baseline frontal QRS-T angle predicted NIMV failure with an AUC of 0.842 (95% CI: 0.773–0.898), using a cut-off of >96° with 76.7% sensitivity and 81.1% specificity. The AUCs were 0.786 (95% CI: 0.708–0.852) for Δ frontal QRS-T angle, 0.788 (95% CI: 0.720–0.846) for baseline pH, 0.765 (95% CI: 0.695–0.824) for PaCO_2_, and 0.746 (95% CI: 0.674–0.807) for lactate. Compared with baseline frontal QRS-T angle, the AUC difference was significant only for lactate (DeLong *p* = 0.046). For in-hospital mortality, baseline frontal QRS-T angle yielded an AUC of 0.811 (95% CI: 0.735–0.873), with 75.9% sensitivity and 78.4% specificity at a cut-off of >104°. The corresponding AUCs were 0.758 (95% CI: 0.678–0.827) for Δ frontal QRS-T angle, 0.779 (95% CI: 0.705–0.840) for baseline pH, 0.754 (95% CI: 0.676–0.820) for PaCO_2_, and 0.792 (95% CI: 0.719–0.851) for lactate. None of the pairwise AUC comparisons with baseline frontal QRS-T angle was statistically significant for in-hospital mortality (all DeLong *p* > 0.05) (Table 7; Figure 3 and Figure 4).

**Figure 5:**
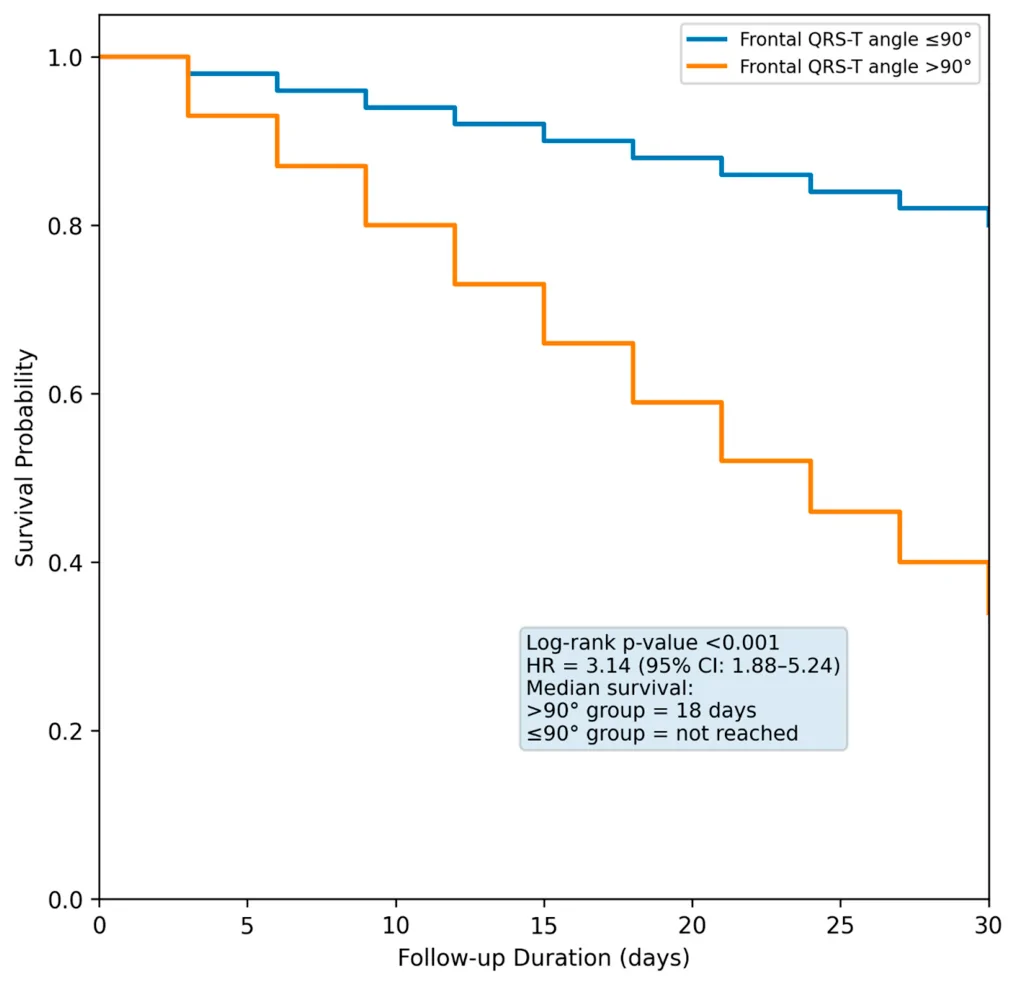
Kaplan–Meier survival curves according to frontal QRS-T angle groups.

Kaplan–Meier survival analysis demonstrated significantly lower survival probabilities in patients with frontal QRS-T angle values >90° compared with patients with frontal QRS-T angle ≤90°. The difference between the survival curves was statistically significant according to the log-rank test (*p* < 0.001). Patients with frontal QRS-T angle >90° had a hazard ratio of 3.14 (95% CI: 1.88–5.24) for in-hospital mortality. Median survival duration in the frontal QRS-T angle >90° group was 18 days, whereas median survival was not reached in patients with frontal QRS-T angle ≤90° during the follow-up period (Figure 5).

Univariate logistic regression analysis demonstrated that advanced age, pneumonia, heart failure, chronic kidney disease, lower baseline pH, elevated PaCO_2_ and lactate levels, increased C-reactive protein, lower serum albumin levels, prolonged QTc interval, higher baseline frontal QRS-T angle, frontal QRS-T angle >90°, reduced Δ frontal QRS-T angle, and need for invasive mechanical ventilation were significantly associated with in-hospital mortality (all *p* < 0.05). In multivariable logistic regression analysis, advanced age (OR: 1.038, 95% CI: 1.006–1.072, *p* = 0.021), pneumonia (OR: 2.148, 95% CI: 1.012–4.562, *p* = 0.046), heart failure (OR: 2.073, 95% CI: 1.011–4.251, *p* = 0.047), chronic kidney disease (OR: 2.441, 95% CI: 1.089–5.471, *p* = 0.030), lower baseline pH (OR: 0.021, 95% CI: 0.001–0.481, *p* = 0.014), higher PaCO_2_ levels (OR: 1.037, 95% CI: 1.006–1.070, *p* = 0.019), elevated lactate levels (OR: 1.468, 95% CI: 1.012–2.129, *p* = 0.043), increased C-reactive protein (OR: 1.051, 95% CI: 1.006–1.099, *p* = 0.026), lower serum albumin levels (OR: 0.411, 95% CI: 0.182–0.927, *p* = 0.032), higher baseline frontal QRS-T angle (OR: 1.254, 95% CI: 1.088–1.446, *p* = 0.002), frontal QRS-T angle >90° (OR: 3.617, 95% CI: 1.484–8.813, *p* = 0.005), smaller Δ frontal QRS-T angle (OR: 0.972, 95% CI: 0.946–0.999, *p* = 0.041), and need for invasive mechanical ventilation (OR: 4.806, 95% CI: 1.824–12.666, *p* = 0.002) remained independent predictors of in-hospital mortality. QTc interval did not retain statistical significance in the multivariable model (*p* = 0.123) (Table 8).

## 4. Discussion

In the present study, we investigated the relationship between dynamic changes in frontal QRS-T angle and treatment response in patients with acute hypercapnic respiratory failure receiving non-invasive mechanical ventilation. Our findings demonstrated that frontal QRS-T angle values significantly decreased after successful NIMV treatment, whereas no significant improvement was observed in patients with NIMV failure. In addition, both baseline frontal QRS-T angle and Δ frontal QRS-T angle showed significant predictive performance for NIMV failure and in-hospital mortality. Changes in frontal QRS-T angle were also significantly correlated with changes in PaCO_2_, pH, and lactate levels following treatment. Furthermore, multivariable logistic regression analysis revealed that frontal QRS-T angle remained an independent predictor of in-hospital mortality even after adjustment for established clinical and biochemical risk factors.

NIMV is considered the standard treatment for acute hypercapnic respiratory failure, particularly in COPD exacerbations accompanied by respiratory acidosis [4,10,11]. Numerous studies have demonstrated that successful NIMV treatment improves gas exchange, reduces intubation rates, shortens hospitalization duration, and decreases mortality [10,11]. However, delayed identification of NIMV failure remains an important clinical problem because prolonged unsuccessful NIMV may increase mortality and worsen organ dysfunction. In our cohort, NIMV failure occurred in 23.1% of patients, while invasive mechanical ventilation was required in 19.9% of the study population. These findings are generally consistent with previous observational studies investigating NIMV outcomes in acute respiratory failure. Hedsund et al. demonstrated that patients requiring NIMV for acute respiratory failure had substantial in-hospital mortality, particularly in the presence of severe hypercapnia and delayed clinical improvement [35]. Abraham et al. reported an NIMV failure rate of approximately 19.8% in patients with acute respiratory failure and demonstrated that unsuccessful NIMV was associated with significantly prolonged hospitalization and increased mortality [12].

Our results demonstrated that patients with failed NIMV treatment had significantly lower baseline pH values and markedly elevated PaCO_2_ levels compared with patients with successful treatment. These findings are compatible with previous studies identifying severe respiratory acidosis and persistent hypercapnia as major predictors of NIMV failure. Confalonieri et al. proposed that worsening acidosis and lack of early physiological response during NIMV represent major determinants of treatment failure in COPD exacerbations [36]. Abraham et al. showed that pH <7.28 and persistent pCO_2_ elevation after NIMV initiation were independently associated with NIMV failure. The strong relationship between persistent hypercapnia and adverse outcomes in our study supports the concept that dynamic physiological recovery is more important than isolated baseline measurements.

The physiological response to NIMV is strongly time-dependent, and clinically meaningful changes in pH, PaCO_2_, respiratory rate, and work of breathing may occur within the first hours of treatment. Because standardized 12-lead ECG recordings were systematically available only at baseline and at 24 ± 2 h in this retrospective cohort, the present study could not determine whether changes in the frontal QRS-T angle emerged at 1, 6, or 12 h or whether they preceded, paralleled, or followed changes in arterial blood gas parameters. Accordingly, the baseline frontal QRS-T angle may be considered an immediately available admission-level risk marker, whereas Δ frontal QRS-T angle at T24 should be interpreted as a marker of physiological response over the first 24 h rather than as a tool for immediate treatment escalation or intubation decisions. Prospective studies should obtain synchronized ECG and arterial blood gas measurements at T0, T1, T6, T12, and T24 and document the exact timing of treatment escalation and endotracheal intubation. In addition, the proposed relationship between frontal QRS-T angle, hypoxemia, pulmonary vascular load, and right ventricular strain remains mechanistic rather than directly demonstrated in this study. Serial echocardiographic parameters, including tricuspid annular plane systolic excursion, right ventricular fractional area change, tissue-Doppler-derived tricuspid annular systolic velocity, and pulmonary artery systolic pressure, were not available. Future prospective studies should evaluate ECG changes together with these right ventricular measurements to determine whether improvement in the frontal QRS-T angle reflects recovery of right ventricular function.

One of the most important findings of our study was the significant reduction in frontal QRS-T angle after successful NIMV treatment. In contrast, patients with failed NIMV treatment did not demonstrate significant improvement in frontal QRS-T angle values. The frontal QRS-T angle reflects ventricular depolarization–repolarization heterogeneity and is considered an indirect marker of myocardial electrical instability [32,33]. Previous studies primarily investigated frontal QRS-T angle in cardiovascular diseases rather than respiratory failure. May et al. reported that enlarged frontal QRS-T angle strongly predicted myocardial infarction and mortality in diabetic patients [29]. Walsh et al. demonstrated that widened frontal QRS-T angle was independently associated with increased all-cause mortality and cardiovascular events in individuals without overt cardiovascular disease [34]. These studies emphasized the prognostic value of frontal QRS-T angle as a marker of electrical and structural myocardial stress. Our study expands these findings by demonstrating that frontal QRS-T angle also dynamically changes in response to correction of acute hypercapnic respiratory failure.

The mechanisms underlying the association between hypercapnic respiratory failure and frontal QRS-T angle abnormalities are likely multifactorial. Acute hypercapnia and hypoxemia can induce pulmonary vasoconstriction, increase right ventricular afterload, and impair myocardial oxygen delivery [13,14,15,16,17,18,19]. Additionally, respiratory acidosis may alter myocardial membrane excitability and repolarization characteristics through changes in intracellular calcium handling and autonomic nervous system activation. As a result, acute respiratory failure may increase ventricular electrical heterogeneity, leading to widening of the frontal QRS-T angle. Successful NIMV treatment may reverse these pathophysiological mechanisms by reducing PaCO_2_, correcting acidosis, improving oxygenation, and decreasing cardiopulmonary stress. The significant correlation observed in our study between ΔPaCO_2_ and Δfrontal QRS-T angle supports this hypothesis.

Another important finding was the significant predictive performance of frontal QRS-T angle for NIMV failure and in-hospital mortality. Baseline frontal QRS-T angle demonstrated an AUC of 0.842 for prediction of NIMV failure and 0.811 for prediction of mortality. In addition, Δ frontal QRS-T angle also showed significant predictive ability for adverse outcomes. These findings suggest that both baseline electrical myocardial stress and its dynamic improvement during treatment are clinically important. Previous studies evaluating predictors of NIMV failure mainly focused on respiratory parameters such as respiratory rate, pH, PaCO_2_, consciousness level, and oxygenation indices [24,25,26]. Our findings indicate that ECG-derived repolarization parameters may provide additional prognostic information beyond traditional respiratory variables.

Kaplan–Meier survival analysis demonstrated significantly lower survival probabilities in patients with frontal QRS-T angle >90°. Furthermore, multivariable logistic regression analysis identified frontal QRS-T angle as an independent predictor of mortality even after adjustment for age, heart failure, chronic kidney disease, pH, lactate, inflammatory markers, and invasive mechanical ventilation requirement. These findings are particularly important because frontal QRS-T angle is inexpensive, non-invasive, rapidly available, and easily reproducible in routine clinical practice. Oehler et al. emphasized that frontal QRS-T angle represents a simple but powerful marker of repolarization heterogeneity associated with arrhythmogenic and cardiovascular risk [32]. Our findings suggest that this parameter may also reflect the severity of systemic physiological stress in acute hypercapnic respiratory failure.

The significant association between elevated frontal QRS-T angle and mortality observed in our study may additionally reflect the high burden of underlying cardiovascular disease in patients with chronic respiratory disorders. Patients with COPD and chronic hypercapnic respiratory failure frequently exhibit pulmonary hypertension, right ventricular dysfunction, systemic inflammation, endothelial dysfunction, and autonomic imbalance [13,14,15,16,17,18,19]. These mechanisms may contribute to ventricular electrical remodeling and explain why frontal QRS-T angle remained independently associated with mortality even after multivariable adjustment.

The findings of this study suggest that the baseline frontal QRS-T angle may provide practical, inexpensive, and readily obtainable admission-level risk information in patients with acute hypercapnic respiratory failure receiving NIMV treatment. Dynamic improvement in frontal QRS-T angle after BPAP/NIMV therapy may reflect successful correction of hypercapnia and cardiopulmonary stress, whereas persistently elevated frontal QRS-T angle values may help identify patients at increased risk for NIMV failure, invasive mechanical ventilation requirement, and in-hospital mortality. Therefore, routine assessment of frontal QRS-T angle during early NIMV follow-up may provide additional prognostic information alongside conventional arterial blood gas and clinical parameters.

Because contemporary digital 12-lead ECG systems automatically generate the frontal QRS and T-wave axes, calculation of the frontal QRS-T angle could potentially be incorporated into ECG software or electronic bedside decision-support systems. Automated serial calculation and trend monitoring might allow clinicians to identify persistent widening or an insufficient reduction in the angle during NIMV and could provide an additional signal of inadequate cardiopulmonary response. However, the present study used two discrete standard 12-lead ECG recordings obtained at T0 and T24 and did not evaluate continuous or real-time angle monitoring. Moreover, axes derived from limited-lead bedside telemetry may not be directly equivalent to those obtained from a standard 12-lead ECG, and measurements may be affected by artifacts, electrode displacement, rhythm changes, or QRS conduction abnormalities. Therefore, the technical feasibility, optimal measurement frequency, alert thresholds, and incremental clinical value of automated real-time frontal QRS-T angle monitoring should be evaluated prospectively before its routine clinical implementation. Such monitoring should be considered an adjunct to, rather than a replacement for, established clinical and arterial blood gas assessment during NIMV.

Our study has several strengths. First, this is, to the best of our knowledge, one of the first studies investigating dynamic changes in frontal QRS-T angle in patients with acute hypercapnic respiratory failure receiving NIMV treatment. Second, we evaluated both baseline and post-treatment ECG measurements together with detailed clinical and arterial blood gas parameters. Third, we combined ROC analysis, survival analysis, and multivariable regression modeling to comprehensively evaluate the prognostic value of frontal QRS-T angle.

Nevertheless, several limitations should be considered. First, the retrospective single-center design may limit the generalizability of the findings and carries an inherent risk of selection bias and residual confounding. Second, standardized 12-lead ECG measurements were systematically available only before NIMV initiation and at 24 ± 2 h. Therefore, we could not evaluate the early temporal trajectory of the frontal QRS-T angle at 1, 6, and 12 h or determine whether its changes preceded, paralleled, or followed the rapidly evolving changes in pH and PaCO_2_. Requiring paired T0–T24 ECG recordings may also have introduced landmark-type selection bias by underrepresenting patients with very early deterioration, intubation, or death before completion of the T24 assessment. Consequently, findings concerning Δ frontal QRS-T angle apply only to patients in whom the scheduled 24-h assessment could be completed and should not be extrapolated to immediate treatment-escalation or intubation decisions. Third, although patients with major ECG confounders such as bundle branch block, pacemaker rhythm, marked QRS prolongation, significant electrolyte abnormalities, and interval rhythm changes were excluded, frontal QRS-T axis measurements may still have been affected by electrode positioning, recording artifacts, medications, or unrecognized structural heart disease. Fourth, serial echocardiographic measurements, including tricuspid annular plane systolic excursion, right ventricular fractional area change, tricuspid annular systolic velocity, and pulmonary artery systolic pressure, were unavailable. Therefore, the proposed relationship between changes in the frontal QRS-T angle and improvement in right ventricular strain remains mechanistic and could not be directly demonstrated. Fifth, differences in the underlying cause of respiratory failure, NIMV settings, concomitant treatments, and cardiovascular comorbidity may have influenced both ECG findings and clinical outcomes despite multivariable adjustment. Finally, long-term outcomes and external validation were unavailable, and the identified prognostic cut-off values were derived from the same cohort. These thresholds should therefore be regarded as exploratory until validated in larger prospective multicenter studies incorporating synchronized ECG, arterial blood gas, and echocardiographic assessments at T0, T1, T6, T12, and T24.

## 5. Conclusions

In conclusion, baseline frontal QRS-T angle was associated with NIMV failure and in-hospital mortality, whereas its change over 24 h was associated with physiological response to treatment. The 24-h change should be interpreted as a response marker rather than an early decision-making tool. Prospective studies incorporating ECG, arterial blood gas, and right ventricular measurements at earlier standardized time points are required before frontal QRS-T angle assessment can be considered for real-time clinical monitoring.

## Figures and Tables

**Table 1:** Demographic, clinical, and outcome characteristics of the overall study population.

Variables	Total Population (*n* = 186)
Age, years	69.4 ± 11.8
Male sex, *n* (%)	118 (63.4)
Body mass index, kg/m^2^	28.1 ± 5.3
Current smoker, *n* (%)	96 (51.6)
Former smoker, *n* (%)	54 (29.0)
**Underlying Respiratory Diseases**	
Chronic obstructive pulmonary disease (COPD), *n* (%)	121 (65.1)
Pneumonia, *n* (%)	38 (20.4)
Bronchiectasis, *n* (%)	14 (7.5)
Interstitial lung disease, *n* (%)	9 (4.8)
Lung malignancy, *n* (%)	11 (5.9)
Chronic respiratory failure, *n* (%)	47 (25.3)
Obesity hypoventilation syndrome, *n* (%)	18 (9.7)
**Comorbid Diseases**	
Hypertension, *n* (%)	102 (54.8)
Diabetes mellitus, *n* (%)	74 (39.8)
Coronary artery disease, *n* (%)	51 (27.4)
Heart failure, *n* (%)	43 (23.1)
Chronic kidney disease, *n* (%)	29 (15.6)
Atrial fibrillation, *n* (%)	17 (9.1)
Cerebrovascular disease, *n* (%)	14 (7.5)
**Vital Signs at Admission**	
Heart rate, beats/min	104 ± 18
Respiratory rate, breaths/min	29 ± 6
Systolic blood pressure, mmHg	132 ± 24
Oxygen saturation (%), room air	82 ± 8
**Clinical Outcomes**	
Successful NIMV treatment, *n* (%)	143 (76.9)
NIMV failure, *n* (%)	43 (23.1)
Need for invasive mechanical ventilation, *n* (%)	37 (19.9)
Intensive care unit stay, days	6 (4–11)
Total hospitalization duration, days	11 (7–17)
In-hospital mortality, *n* (%)	29 (15.6)

NIMV, non-invasive mechanical ventilation.

**Table 2:** Baseline arterial blood gas, laboratory, and electrocardiographic characteristics of the overall study population.

Variables	Total Population (*n* = 186)
**Arterial Blood Gas Parameters**	
pH	7.28 ± 0.07
PaCO_2_, mmHg	68.9 ± 14.6
PaO_2_, mmHg	58.4 ± 12.7
HCO_3_^−^, mmol/L	31.6 ± 6.5
Lactate, mmol/L	2.3 ± 1.1
**Laboratory Parameters**	
White blood cell count, ×10^3^/µL	11.8 ± 4.7
Hemoglobin, g/dL	13.1 ± 2.0
Lymphocyte percentage, %	15.4 ± 8.2
Platelet count, ×10^3^/µL	256 ± 88
C-reactive protein, mg/L	48 (22–96)
Serum albumin, g/dL	3.4 ± 0.6
Glucose, mg/dL	162 ± 58
Creatinine, mg/dL	1.12 ± 0.48
**Electrocardiographic Parameters**	
Baseline sinus rhythm, *n* (%)	169 (90.9)
Heart rate on ECG, beats/min	101 ± 17
QRS duration, ms	96 ± 11
QTc interval, ms	438 ± 29
Baseline frontal QRS-T angle, degrees	82 (54–118)
Frontal QRS-T angle > 90°, *n* (%)	79 (42.5)

ECG, electrocardiography; HCO_3_^−^, bicarbonate; PaCO_2_, partial arterial carbon dioxide pressure; PaO_2_, partial arterial oxygen pressure; QTc, corrected QT interval.

**Table 3:** Comparison of demographic and clinical characteristics according to NIMV outcome.

Variables	Successful NIMV (*n* = 143)	Failed NIMV (*n* = 43)	*p*-Value
Age, years	67.8 ± 10.9	74.2 ± 12.4	0.002
Male sex, *n* (%)	88 (61.5)	30 (69.8)	0.326
Body mass index, kg/m^2^	28.5 ± 5.1	26.9 ± 5.8	0.084
Current smoker, *n* (%)	69 (48.3)	27 (62.8)	0.091
**Underlying Respiratory Diseases**			
COPD, *n* (%)	91 (63.6)	30 (69.8)	0.461
Pneumonia, *n* (%)	24 (16.8)	14 (32.6)	0.024
Bronchiectasis, *n* (%)	12 (8.4)	2 (4.7)	0.531
Interstitial lung disease, *n* (%)	5 (3.5)	4 (9.3)	0.124
Lung malignancy, *n* (%)	6 (4.2)	5 (11.6)	0.067
Obesity hypoventilation syndrome, *n* (%)	16 (11.2)	2 (4.7)	0.247
**Comorbid Diseases**			
Hypertension, *n* (%)	74 (51.7)	28 (65.1)	0.121
Diabetes mellitus, *n* (%)	52 (36.4)	22 (51.2)	0.083
Coronary artery disease, *n* (%)	34 (23.8)	17 (39.5)	0.045
Heart failure, *n* (%)	27 (18.9)	16 (37.2)	0.011
Chronic kidney disease, *n* (%)	17 (11.9)	12 (27.9)	0.014
**Vital Signs at Admission**			
Heart rate, beats/min	101 ± 17	112 ± 19	0.001
Respiratory rate, breaths/min	28 ± 5	32 ± 7	<0.001
Oxygen saturation (%), room air	84 ± 7	76 ± 8	<0.001

Data are presented as mean ± standard deviation or number (%).

**Table 4:** Comparison of arterial blood gas, laboratory, electrocardiographic, and clinical outcome variables according to NIMV outcome.

Variables	Successful NIMV (*n* = 143)	Failed NIMV (*n* = 43)	*p*-Value
**Arterial Blood Gas Parameters**			
pH	7.30 ± 0.06	7.22 ± 0.08	<0.001
PaCO_2_, mmHg	65.4 ± 12.2	80.1 ± 16.3	<0.001
PaO_2_, mmHg	60.2 ± 11.4	52.1 ± 13.5	<0.001
HCO_3_^−^, mmol/L	30.8 ± 5.8	34.1 ± 7.2	0.003
Lactate, mmol/L	2.0 ± 0.9	3.1 ± 1.4	<0.001
**Laboratory Parameters**			
White blood cell count, ×10^3^/µL	10.9 ± 4.2	14.8 ± 5.1	<0.001
Hemoglobin, g/dL	13.3 ± 1.9	12.5 ± 2.2	0.031
Lymphocyte percentage, %	16.8 ± 7.9	10.7 ± 6.2	<0.001
Platelet count, ×10^3^/µL	262 ± 84	238 ± 91	0.118
C-reactive protein, mg/L	39 (18–72)	91 (48–156)	<0.001
Serum albumin, g/dL	3.5 ± 0.5	3.0 ± 0.6	<0.001
Glucose, mg/dL	154 ± 51	188 ± 69	0.002
Creatinine, mg/dL	1.04 ± 0.39	1.39 ± 0.62	<0.001
**Electrocardiographic Parameters**			
Heart rate on ECG, beats/min	99 ± 16	110 ± 18	<0.001
QRS duration, ms	95 ± 10	101 ± 13	0.002
QTc interval, ms	432 ± 26	457 ± 34	<0.001
Baseline frontal QRS-T angle, degrees	71 (48–101)	128 (94–156)	<0.001
Frontal QRS-T angle >90°, *n* (%)	46 (32.2)	33 (76.7)	<0.001
Δ Frontal QRS-T angle, degrees	−24 (−41 to −10)	−5 (−12 to 4)	<0.001
**Clinical Outcomes**			
Need for invasive mechanical ventilation, *n* (%)	0 (0)	37 (86.0)	<0.001
Intensive care unit stay, days	5 (3–8)	14 (9–21)	<0.001
Total hospitalization duration, days	9 (6–13)	19 (12–28)	<0.001
In-hospital mortality, *n* (%)	9 (6.3)	20 (46.5)	<0.001

Data are presented as mean ± standard deviation, median (interquartile range), or number (%). COPD, chronic obstructive pulmonary disease; ECG, electrocardiography; HCO_3_^−^, bicarbonate; NIMV, non-invasive mechanical ventilation; PaCO_2_, partial arterial carbon dioxide pressure; PaO_2_, partial arterial oxygen pressure; QTc, corrected QT interval.

**Table 5:** Comparison of pre-treatment and post-treatment frontal QRS-T angle measurements.

Electrocardiographic Parameters	Pre-Treatment (T0)	Post-Treatment (T24)	*p*-Value
**Overall Study Population (*n* = 186)**			
Frontal QRS-T angle, degrees	82 (54–118)	61 (39–92)	<0.001
Heart rate on ECG, beats/min	101 ± 17	92 ± 15	<0.001
QRS duration, ms	96 ± 11	94 ± 10	0.041
QTc interval, ms	438 ± 29	425 ± 24	<0.001
**Successful NIMV Group (*n* = 143)**			
Frontal QRS-T angle, degrees	71 (48–101)	44 (28–73)	<0.001
Heart rate on ECG, beats/min	99 ± 16	88 ± 13	<0.001
QRS duration, ms	95 ± 10	93 ± 9	0.032
QTc interval, ms	432 ± 26	419 ± 21	<0.001
**Failed NIMV Group (*n* = 43)**			
Frontal QRS-T angle, degrees	128 (94–156)	119 (88–149)	0.071
Heart rate on ECG, beats/min	110 ± 18	105 ± 17	0.084
QRS duration, ms	101 ± 13	100 ± 12	0.412
QTc interval, ms	457 ± 34	451 ± 31	0.118

Data are presented as mean ± standard deviation or median (interquartile range). ECG, electrocardiography; NIMV, non-invasive mechanical ventilation; QTc, corrected QT interval. T0: immediately before initiation of BPAP/NIMV treatment. T24: 24th ± 2 h of treatment.

**Table 6:** Correlation analysis between changes in PaCO_2_ levels and frontal QRS-T angle values.

Variables	Correlation Coefficient (r)	*p*-Value
ΔPaCO_2_ vs. ΔFrontal QRS-T angle	0.624	<0.001
ΔPaCO_2_ vs. ΔQTc interval	0.381	<0.001
ΔPaCO_2_ vs. ΔHeart rate	0.294	0.002
ΔpH vs. ΔFrontal QRS-T angle	−0.517	<0.001
ΔPaO_2_ vs. ΔFrontal QRS-T angle	−0.248	0.011
ΔLactate vs. ΔFrontal QRS-T angle	0.336	<0.001

PaCO_2_, partial arterial carbon dioxide pressure; PaO_2_, partial arterial oxygen pressure; QTc, corrected QT interval. Correlation analysis was performed using Pearson or Spearman correlation tests according to variable distribution characteristics. Δ (delta) values represent the difference between baseline (T0) and 24-h post-treatment (T24) measurements.

**Table 7:** Comparative ROC curve analysis of frontal QRS-T angle and established physiological parameters for predicting NIMV failure and in-hospital mortality.

Outcome and Predictor	Optimal Cut-Off	AUC (95% CI)	*p*-Value	Sensitivity (%)	Specificity (%)	PPV (%)	NPV (%)	DeLong *p*-Value *
**NIMV failure**								
Baseline frontal QRS-T angle	>96°	0.842 (0.773–0.898)	<0.001	76.7	81.1	55.0	92.1	Reference
Δ frontal QRS-T angle	>−12°	0.786 (0.708–0.852)	<0.001	72.1	74.8	46.3	89.9	0.184
Baseline pH	≤7.25	0.788 (0.720–0.846)	<0.001	69.8	76.2	46.9	89.3	0.327
Baseline PaCO_2_	>72 mmHg	0.765 (0.695–0.824)	<0.001	72.1	70.6	42.5	89.4	0.118
Baseline lactate	>2.4 mmol/L	0.746 (0.674–0.807)	<0.001	67.4	72.0	42.0	88.0	0.046
**In-hospital mortality**								
Baseline frontal QRS-T angle	>104°	0.811 (0.735–0.873)	<0.001	75.9	78.4	39.3	94.6	Reference
Δ frontal QRS-T angle	>−8°	0.758 (0.678–0.827)	0.002	69.0	73.5	32.5	92.8	0.286
Baseline pH	≤7.24	0.779 (0.705–0.840)	<0.001	72.4	70.7	31.3	93.3	0.534
Baseline PaCO_2_	>74 mmHg	0.754 (0.676–0.820)	<0.001	69.0	68.8	29.0	92.3	0.214
Baseline lactate	>2.6 mmol/L	0.792 (0.719–0.851)	<0.001	72.4	75.2	35.0	93.7	0.682

Data are presented as area under the curve with 95% confidence intervals. AUC, area under the curve; CI, confidence interval; NIMV, non-invasive mechanical ventilation; NPV, negative predictive value; PaCO_2_, partial arterial carbon dioxide pressure; PPV, positive predictive value. Optimal cut-off values were determined using the Youden index. * Pairwise comparisons were performed against the AUC of baseline frontal QRS-T angle using DeLong’s test. Δ frontal QRS-T angle was calculated as T24 minus T0; therefore, values above the specified negative thresholds indicate less angle narrowing and greater risk.

**Table 8:** Univariable and multivariable logistic regression analyses for in-hospital mortality.

Variables	Univariate OR (95% CI)	*p*-Value	Multivariable OR (95% CI)	*p*-Value
Age (per 1-year increase)	1.052 (1.021–1.085)	0.001	1.038 (1.006–1.072)	0.021
Male sex	1.294 (0.612–2.739)	0.499	—	—
COPD	1.183 (0.542–2.584)	0.672	—	—
Pneumonia	2.964 (1.337–6.571)	0.007	2.148 (1.012–4.562)	0.046
Heart failure	3.121 (1.449–6.724)	0.004	2.073 (1.011–4.251)	0.047
Chronic kidney disease	3.844 (1.693–8.728)	0.001	2.441 (1.089–5.471)	0.030
Baseline pH	0.001 (0.000–0.047)	<0.001	0.021 (0.001–0.481)	0.014
PaCO_2_ (per 1 mmHg increase)	1.058 (1.028–1.089)	<0.001	1.037 (1.006–1.070)	0.019
Lactate (per 1 mmol/L increase)	1.842 (1.301–2.608)	<0.001	1.468 (1.012–2.129)	0.043
C-reactive protein (per 10 mg/L increase)	1.087 (1.032–1.145)	0.002	1.051 (1.006–1.099)	0.026
Serum albumin (per 1 g/dL increase)	0.284 (0.134–0.603)	0.001	0.411 (0.182–0.927)	0.032
QTc interval (per 10 ms increase)	1.219 (1.086–1.368)	0.001	1.108 (0.972–1.264)	0.123
Baseline frontal QRS-T angle (per 10° increase)	1.371 (1.205–1.559)	<0.001	1.254 (1.088–1.446)	0.002
Frontal QRS-T angle > 90°	5.928 (2.561–13.718)	<0.001	3.617 (1.484–8.813)	0.005
Δ Frontal QRS-T angle	0.958 (0.934–0.982)	0.001	0.972 (0.946–0.999)	0.041
Need for invasive mechanical ventilation	8.491 (3.548–20.321)	<0.001	4.806 (1.824–12.666)	0.002

## Data Availability

The original contributions presented in this study are included in the article. Further inquiries can be directed to the corresponding author.

## Abbreviations

AUC	Area under the curve
BPAP	Bilevel positive airway pressure
CI	Confidence interval
COPD	Chronic obstructive pulmonary disease
CRP	C-reactive protein
ECG	Electrocardiography
HCO_3_^−^	Bicarbonate
ICU	Intensive care unit
IQR	Interquartile range
LBBB	Left bundle branch block
NIMV	Non-invasive mechanical ventilation
NIV	Non-invasive ventilation
NPV	Negative predictive value
OR	Odds ratio
PaCO_2_	Partial arterial carbon dioxide pressure
PaO_2_	Partial arterial oxygen pressure
PPV	Positive predictive value
QTc	Corrected QT interval
ROC	Receiver operating characteristic
T0	Baseline measurement before NIMV treatment
T24	Measurement at the 24th hour of NIMV treatment
ΔQRS-T angle	Change in frontal QRS-T angle
